# Dental students’ perceptions of preclinical and clinical prosthodontic education: a cross-sectional survey

**DOI:** 10.1186/s12909-026-09751-1

**Published:** 2026-07-08

**Authors:** Hamiyet Güngör, Ceren Karaahmetoglu

**Affiliations:** Department of Prosthodontics, Faculty of Dentistry, Lokman Hekim University, Söğütözü Dist. 2179 St.No:4, Çankaya, Ankara, 06510 Turkey

**Keywords:** Prosthodontics, Dental education, Clinical competence, Dental students

## Abstract

**Background:**

This study aimed to evaluate the relationship between preclinical and clinical prosthodontic training and self-perceived clinical competence among 4th- and 5th-year dental students at Lokman Hekim University Faculty of Dentistry.

**Methods:**

Ethical approval was obtained prior to the study (Decision No: 2025/5; Protocol No: 2025127). A 24-item questionnaire using five-point Likert-scale responses was administered to 131 4th- and 5th-year dental students during the 2024–2025 academic year. The questionnaire included three domains: preclinical-clinical practice similarity and self-perceived clinical competence, curriculum and educational content, and clinical confidence and learning needs. The instrument was developed based on relevant literature, reviewed by prosthodontic experts, and pilot tested before administration. Internal consistency was assessed using Cronbach’s alpha. Data were analyzed using SPSS 27 software; Chi-square, Fisher’s exact, and Mann–Whitney U tests were used, with Bonferroni correction applied for multiple comparisons.

**Results:**

Significant differences were observed according to academic year and gender. 5th-year students reported lower perceived sufficiency of preclinical training on impression procedures for fixed prostheses *(p* = 0.017), more frequent experience with virtual reality applications (*p* < 0.001), higher self-perceived knowledge in selected removable partial denture procedures (*p* = 0.015), and a higher perceived need for increased preclinical training (*p* = 0.028). Male students reported higher self-confidence during clinical practice than female students (*p* = 0.008). Significant associations were also found between perceived alignment of preclinical-clinical practices with current treatment protocols, perceived need for curriculum revision, and self-perceived clinical competence (*p* < 0.001). Effect size analysis showed small-to-moderate effects for most significant findings, with the largest effect observed for virtual reality use according to academic year (*r* = 0.584).

**Conclusion:**

The findings suggest that students’ perceptions of prosthodontic education vary according to academic year and gender. Since the study was based on self-reported questionnaire data, the findings reflect self-perceived clinical competence rather than objectively measured clinical competence. Closer alignment between preclinical and clinical instruction, integration of updated clinical procedures, and expanded early clinical exposure may support students’ perceived preparedness and clinical confidence.

**Supplementary Information:**

The online version contains supplementary material available at https://doi.org/10.1186/s12909-026-09751-1.

## Background

Dental education provides a progressive learning process in which students first acquire fundamental theoretical knowledge, followed by preclinical exercises and clinical patient-based training [1]. In dental schools, students develop their basic manual skills through preclinical practices during the first three years of education, and in the final two years, they participate directly in patient treatment in the clinical setting, reinforcing their knowledge and skills through real patient experiences [2]. In particular, prosthodontics education in dental faculties is a discipline that requires the integration of theoretical knowledge, laboratory procedures, manual skills, clinical decision-making, and patient-centered treatment planning [3]. Prosthodontics involves the diagnosis, treatment planning, maintenance, and rehabilitation of complex clinical conditions, which provides a clear rationale for evaluating this discipline specifically rather than dental education in general [4]. Its educational content requires students to integrate material selection, esthetic and occlusal assessment, laboratory communication, and management of patient expectations. Within the Turkish undergraduate dental curriculum context, preclinical prosthodontic training generally introduces these concepts through phantom model and laboratory-based exercises, including fixed and removable prosthodontic procedures, tooth preparation, impression techniques, occlusion, shade selection, and laboratory workflows. Clinical training then extends these skills to patient-based settings through examination, diagnosis, treatment planning, prosthodontic treatment procedures, esthetic and occlusal evaluation, and supervised patient management.

In prosthodontics education, the preclinical phase allows students to develop an abstract understanding of fundamental clinical procedures [3, 5]. However, previous studies have reported that students often face difficulties in transferring theoretical and practical knowledge gained during preclinical training into clinical practice; in particular, the lack of hands-on experience negatively affects students’ confidence and their perceived clinical competence [6, 7]. It has been emphasized that preclinical training should be redesigned to directly align with clinical procedures and supported by modern teaching approaches such as simulation and video demonstrations [3, 8].

Students’ levels of confidence in clinical practice depend not only on their theoretical knowledge but also on the extent of practical training and the quality of instruction that support students’ perceived clinical competence [1, 7]. The literature indicates that early exposure to clinical procedures during dental education may enhance students’ self-confidence and contribute to greater clinical readiness [9, 10]. Moreover, incorporating updated curricula, including selected digital dentistry applications, may facilitate students’ adaptation to contemporary clinical practice [11].

Recent studies have demonstrated that improving both the theoretical and clinical components of prosthodontic education may influence students’ perceived preparedness for professional practice after graduation [6, 7]. Considering the complexity and multi-step nature of prosthodontic procedures, it is essential that students receive sufficient hands-on experience during the preclinical phase and gain confidence in patient management during clinical practice [1].

Although previous studies have evaluated dental students’ perceptions of prosthodontic education, limited evidence is available on how students at different stages of clinical training perceive the relationship between preclinical-clinical training alignment, clinical confidence, learning needs, and self-perceived competence. In this context, the aim of the present study was to evaluate the relationship between theoretical and practical training in prosthodontics and self-perceived clinical competence among 4th- and 5th-year dental students at Lokman Hekim University Faculty of Dentistry. The null hypotheses were as follows: (1) students’ perceptions of prosthodontic education, self-reported clinical confidence, learning needs, and self-perceived clinical competence would not differ significantly according to academic year or gender; and (2) there would be no significant association between students’ perceptions of preclinical-clinical training alignment and their perceived need for curriculum revision, self-reported clinical confidence, or self-perceived clinical competence.

## Methods

Ethical approval was obtained from the Scientific Research Ethics Committee of the Faculty of Dentistry at Lokman Hekim University prior to study initiation (Decision No: 2025/5; Protocol No: 2025127). All procedures involving human participants were conducted in accordance with the ethical principles of the Declaration of Helsinki and complied with relevant national regulations.

This cross-sectional study utilized a 24-item questionnaire administered to 4th- and 5th-year dental students at the Faculty of Dentistry, Lokman Hekim University. All 4th- and 5th-year dental students enrolled during the 2024–2025 academic year were invited to participate in the study. A total of 150 students were eligible, and 131 students completed the questionnaire, yielding a response rate of 87.3%. Participation was voluntary. The minimum required sample size was estimated as 109 students using a finite population sample size calculation with a 95% confidence level, a 5% margin of error, and a 50% response distribution. Therefore, the final sample size exceeded the minimum required sample size.

The questionnaire was specifically developed for this study to evaluate dental students’ perceptions of the preclinical and clinical prosthodontic curriculum and was administered using the Google Forms online survey tool. The introductory section of the form provided information about the purpose, scope, and ethical aspects of the study. Electronic informed consent was obtained from all participants prior to participation. Responses were anonymous to the researchers; no personally identifiable information, such as name, email address, or student ID, was collected at any stage, and participant confidentiality was strictly maintained throughout the data collection process. To minimize duplicate submissions, the “Limit to 1 response” setting was enabled in Google Forms. Although this required respondents to sign in with a Google account, email addresses were not recorded in the response dataset or accessible to the researchers. The first section of the questionnaire included demographic variables such as age, gender, and academic year. The subsequent sections were structured under three main domains: preclinical-clinical practice similarity and self-perceived clinical competence (5 items), curriculum and educational content (14 items), and clinical confidence and learning needs (5 items).

All questionnaire items were rated on a five-point Likert scale ranging from “strongly disagree” to “strongly agree” and were coded from 1 to 5 for statistical analysis. Since responses to all items were mandatory in Google Forms, no missing data were present in the final dataset.

The questionnaire items were generated based on a comprehensive review of previously published survey studies focusing on prosthodontic education, preclinical and clinical training, curriculum content, teaching methods, and students’ self-perceived clinical competence. While the questionnaire was not directly adopted from a single previously validated instrument, similar established studies were reviewed during item generation to ensure alignment with the primary objectives of the present study.

To ensure content validity, the draft questionnaire was evaluated by a panel of prosthodontic experts for content relevance, clarity, and comprehensibility, and minor revisions were made based on their feedback. Subsequently, a pilot test was conducted with 25 participants to evaluate the practicability, clarity, and interpretability of the items. The participants included in the pilot test were not included in the final study sample. Final modifications were made according to the feedback obtained from the pilot study.

To assess the internal consistency and reliability of the questionnaire, Cronbach’s alpha coefficient was calculated. The overall questionnaire demonstrated good internal consistency (α = 0.864). For the subscales, Cronbach’s alpha values were 0.712 for items related to preclinical-clinical practice similarity and self-perceived clinical competence, 0.814 for curriculum and educational content, and 0.704 for clinical confidence and learning needs, indicating acceptable to good internal consistency across the questionnaire domains. The content validity of the questionnaire was evaluated using the Lawshe method. Based on the assessments of five experts, all questionnaire items were rated as essential, and the content validity value was calculated as 1.00. Since this value exceeded the minimum acceptable threshold, no items required exclusion. The full English version of the questionnaire is provided as Supplementary Material (Supplementary Material 1). Data were analyzed using SPSS version 27 software. Descriptive statistics were presented as frequencies and percentages. Relationships between categorical variables and associations between questionnaire items were examined using the Chi-square test and Fisher’s exact test. Mann–Whitney U tests were additionally performed to compare responses according to academic year and gender. To adjust for multiple comparisons, Bonferroni correction was applied separately for academic year and gender comparisons. Effect sizes were calculated using Cramer’s V for Chi-square and Fisher’s exact tests and r values for Mann-Whitney U tests to support the interpretation of practical significance. A significance level of α = 0.05 was used for all statistical analyses.

## Results

A total of 131 dental students participated in the study, including 87 females (66.4%) and 44 males (33.6%), with representation from the 4th (50.4%) and 5th (49.6%) academic years. In the analysis of questions related to preclinical-clinical practice similarity and self-perceived clinical competence, statistically significant differences were found according to both academic year and gender variables, as presented in Table 1. Fifth-year students reported greater difficulty in performing preclinical practical assignments than 4th-year students (*p* = 0.041). Regarding gender, male students more frequently perceived preclinical exercises as similar to current treatment procedures (*p* = 0.046), whereas female students more frequently reported the need for revising the preclinical and clinical curriculum according to current treatment procedures (*p* = 0.006).

**Table 1:** Comparison of students’ responses to the questionnaire items related to preclinical-clinical practice similarity and self-perceived clinical competence according to academic year and gender

Questions	Responses	4th-year	5th-year	*p*	Female	Male	*p*
		*n* (%)	*n* (%)		*n* (%)	*n* (%)	
I had difficulty performing the practical assignments given in the preclinical prosthodontics courses.	Strongly disagree	11 (16.7)	4 (6.2)	0.041*	9 (10.3)	6 (13.6)	0.607
	Disagree	22 (33.3)	18 (27.7)		26 (29.9)	14 (31.8)	
	Neutral	25 (37.9)	23 (35.4)		34 (39.1)	14 (31.8)	
	Agree	8 (12.1)	17 (26.2)		15 (17.2)	10 (22.7)	
	Strongly agree	0 (0)	3 (4.6)		3 (3.4)	0 (0)	
The procedures performed in the preclinical courses are similar to current treatment protocols.	Strongly disagree	2 (3)	6 (9.2)	0.246	3 (3.4)	5 (11.4)	0.046*
	Disagree	11 (16.7)	12 (18.5)		16 (18.4)	7 (15.9)	
	Neutral	19 (28.8)	24 (36.9)		34 (39.1)	9 (20.5)	
	Agree	27 (40.9)	16 (24.6)		28 (32.2)	15 (34.1)	
	Strongly agree	7 (10.6)	7 (10.8)		6 (6.9)	8 (18.2)	
The procedures performed in the clinical practice are similar to current treatment protocols.	Strongly disagree	2 (3)	3 (4.6)	0.581	2 (2.3)	3 (6.8)	0.277
	Disagree	6 (9.1)	9 (13.8)		12 (13.8)	3 (6.8)	
	Neutral	20 (30.3)	25 (38.5)		32 (36.8)	13 (29.5)	
	Agree	26 (39.4)	19 (29.2)		30 (34.5)	15 (34.1)	
	Strongly agree	12 (18.2)	9 (13.8)		11 (12.6)	10 (22.7)	
I believe that the preclinical and clinical curriculum needs to be revised according to current treatment protocols.	Strongly disagree	4 (6.1)	2 (3.1)	0.214	0 (0)	6 (13.6)	0.006*
	Disagree	13 (19.7)	8 (12.3)		12 (13.8)	9 (20.5)	
	Neutral	18 (27.3)	20 (30.8)		27 (31)	11 (25)	
	Agree	23 (34.8)	18 (27.7)		31 (35.6)	10 (22.7)	
	Strongly agree	8 (12.1)	17 (26.2)		17 (19.5)	8 (18.2)	
I believe that the current clinical training is sufficient for professional practice after graduation.	Strongly disagree	7 (10.6)	5 (7.7)	0.242	8 (9.2)	4 (9.1)	0.482
	Disagree	13 (19.7)	22 (33.8)		26 (29.9)	9 (20.5)	
	Neutral	27 (40.9)	22 (33.8)		34 (39.1)	15 (34.1)	
	Agree	15 (22.7)	9 (13.8)		13 (14.9)	11 (25)	
	Strongly agree	4 (6.1)	7 (10.8)		6 (6.9)	5 (11.4)	

(n: frequency; %: percentage). The statistically significant *p* values are shown in bold (*p < 0.05)

In the questions related to curriculum and educational content, statistically significant differences were observed for selected items according to academic year and gender, as shown in Table 2. When evaluated by academic year, 5th-year students were more likely to report insufficient preclinical training on impression procedures for fixed prostheses (*p* = 0.004) and greater experience with virtual reality applications (*p* < 0.001). Fourth-year students more frequently reported that preclinical education contributed to the development of their aesthetic perception (*p* = 0.032). Fifth-year students also reported higher self-perceived knowledge regarding removable partial denture fabrication (*p* = 0.004).

**Table 2:** Comparison of students’ responses to the questionnaire items related to curriculum and educational content according to academic year and gender

Questions	Responses	4th-year	5th-year	*p*	Female	Male	*p*
		*n* (%)	*n* (%)		*n* (%)	*n* (%)	
The fixed prosthodontic procedures performed in preclinical courses are similar to those performed in clinical practice.	Strongly disagree	2 (3)	9 (13.8)	0.196	6 (6.9)	5 (11.4)	0.079
	Disagree	12 (18.2)	7 (10.8)		16 (18.4)	3 (6.8)	
	Neutral	25 (37.9)	23 (35.4)		34 (39.1)	14 (31.8)	
	Agree	23 (34.8)	21 (32.3)		28 (32.2)	16 (36.4)	
	Strongly agree	4 (6.1)	5 (7.7)		3 (3.4)	6 (13.6)	
The preclinical courses on tooth preparation procedures for fixed prostheses are sufficient.	Strongly disagree	3 (4.5)	3 (4.6)	0.160	1 (1.1)	5 (11.4)	0.024*
	Disagree	2 (3)	9 (13.8)		6 (6.9)	5 (11.4)	
	Neutral	19 (28.8)	22 (33.8)		31 (35.6)	10 (22.7)	
	Agree	32 (48.5)	22 (33.8)		39 (44.8)	15 (34.1)	
	Strongly agree	10 (15.2)	9 (13.8)		10 (11.5)	9 (20.5)	
The preclinical courses on impression procedures for fixed prostheses are sufficient.	Strongly disagree	6 (9.1)	18 (27.7)	0.004*	16 (18.4)	8 (18.2)	0.008*
	Disagree	7 (10.6)	14 (21.5)		18 (20.7)	3 (6.8)	
	Neutral	23 (34.8)	18 (27.7)		29 (33.3)	12 (27.3)	
	Agree	25 (37.9)	10 (15.4)		22 (25.3)	13 (29.5)	
	Strongly agree	5 (7.6)	5 (7.7)		2 (2.3)	8 (18.2)	
The clinical procedures for fixed prosthodontic treatments are similar to current treatment protocols.	Strongly disagree	0 (0)	4 (6.2)	0.197	2 (2.3)	2 (4.5)	0.099
	Disagree	8 (12.1)	9 (13.8)		13 (14.9)	4 (9.1)	
	Neutral	28 (42.4)	31 (47.7)		42 (48.3)	17 (38.6)	
	Agree	25 (37.9)	16 (24.6)		27 (31)	14 (31.8)	
	Strongly agree	5 (7.6)	5 (7.7)		3 (3.4)	7 (15.9)	
I have received adequate theoretical training on digital impression and workflow.	Strongly disagree	10 (15.2)	9 (13.8)	0.423	15 (17.2)	4 (9.1)	0.389
	Disagree	13 (19.7)	19 (29.2)		22 (25.3)	10 (22.7)	
	Neutral	25 (37.9)	17 (26.2)		26 (29.9)	16 (36.4)	
	Agree	12 (18.2)	10 (15.4)		16 (18.4)	6 (13.6)	
	Strongly agree	6 (9.1)	10 (15.4)		8 (9.2)	8 (18.2)	
I have had experience with digital impression procedures during clinical practice.	Strongly disagree	33 (50)	38 (58.5)	0.183	53 (60.9)	18 (40.9)	0.157
	Disagree	11 (16.7)	7 (10.8)		12 (13.8)	6 (13.6)	
	Neutral	14 (21.2)	9 (13.8)		11 (12.6)	12 (27.3)	
	Agree	7 (10.6)	5 (7.7)		7 (8)	5 (11.4)	
	Strongly agree	1 (1.5)	6 (9.2)		4 (4.6)	3 (6.8)	
I have used virtual reality applications.	Strongly disagree	47 (71.2)	12 (18.5)	< 0.001*	40 (46)	19 (43.2)	0.371
	Disagree	6 (9.1)	10 (15.4)		10 (11.5)	6 (13.6)	
	Neutral	11 (16.7)	9 (13.8)		14 (16.1)	6 (13.6)	
	Agree	1 (1.5)	15 (23.1)		13 (14.9)	3 (6.8)	
	Strongly agree	1 (1.5)	19 (29.2)		10 (11.5)	10 (22.7)	
I have found the use of virtual reality applications beneficial for chairside procedures.	Strongly disagree	38 (57.6)	36 (55.4)	0.250	46 (52.9)	28 (63.6)	0.701
	Disagree	9 (13.6)	12 (18.5)		15 (17.2)	6 (13.6)	
	Neutral	16 (24.2)	11 (16.9)		20 (23)	7 (15.9)	
	Agree	3 (4.5)	2 (3.1)		4 (4.6)	1 (2.3)	
	Strongly agree	0 (0)a	4 (6.2)		2 (2.3)	2 (4.5)	
I have sufficient knowledge about shade selection in clinical practice.	Strongly disagree	2 (3)	2 (3.1)	0.646	2 (2.3)	2 (4.5)	0.673
	Disagree	5 (7.6)	7 (10.8)		9 (10.3)	3 (6.8)	
	Neutral	17 (25.8)	15 (23.1)		20 (23)	12 (27.3)	
	Agree	27 (40.9)	20 (30.8)		34 (39.1)	13 (29.5)	
	Strongly agree	15 (22.7)	21 (32.3)		22 (25.3)	14 (31.8)	
Preclinical education has contributed to the development of my aesthetic perception.	Strongly disagree	1 (1.5)	7 (10.8)	0.032*	4 (4.6)	4 (9.1)	0.406
	Disagree	5 (7.6)	11 (16.9)		10 (11.5)	6 (13.6)	
	Neutral	19 (28.8)	18 (27.7)		25 (28.7)	12 (27.3)	
	Agree	29 (43.9)	16 (24.6)		34 (39.1)	11 (25)	
	Strongly agree	12 (18.2)	13 (20)		14 (16.1)	11 (25)	
The removable partial denture procedures performed in preclinical courses are similar to those performed in clinical practice.	Strongly disagree	5 (7.6)	11 (16.9)	0.215	12 (13.8)	4 (9.1)	0.059
	Disagree	17 (25.8)	12 (18.5)		23 (26.4)	6 (13.6)	
	Neutral	23 (34.8)	15 (23.1)		26 (29.9)	12 (27.3)	
	Agree	15 (22.7)	21 (32.3)		22 (25.3)	14 (31.8)	
	Strongly agree	6 (9.1)	6 (9.2)		4 (4.6)	8 (18.2)	
I have sufficient knowledge to plan appropriate treatments and fabricate removable partial dentures for partially edentulous patients.	Strongly disagree	3 (4.5)	2 (3.1)	0.149	3 (3.4)	2 (4.5)	0.174
	Disagree	7 (10.6)	6 (9.2)		9 (10.3)	4 (9.1)	
	Neutral	28 (42.4)	16 (24.6)		33 (37.9)	11 (25)	
	Agree	20 (30.3)	25 (38.5)		31 (35.6)	14 (31.8)	
	Strongly agree	8 (12.1)	16 (24.6)		11 (12.6)	13 (29.5)	
I have sufficient knowledge to perform the necessary intraoral procedures, obtain ideal impressions and occlusion, and achieve esthetics in removable partial denture fabrication.	Strongly disagree	0 (0)	1 (1.5)	0.004*	1 (1.1)	0 (0)	< 0.001*
	Disagree	12 (18.2)	6 (9.2)		13 (14.9)	5 (11.4)	
	Neutral	32 (48.5)	17 (26.2)		36 (41.4)	13 (29.5)	
	Agree	19 (28.8)	28 (43.1)		34 (39.1)	13 (29.5)	
	Strongly agree	3 (4.5)	13 (20)		3 (3.4)	13 (29.5)	
The complete denture procedures performed in preclinical courses are similar to those performed in clinical practice.	Strongly disagree	2 (3)	7 (10.8)	0.175	7 (8)	2 (4.5)	0.096
	Disagree	12 (18.2)	5 (7.7)		14 (16.1)	3 (6.8)	
	Neutral	25 (37.9)	22 (33.8)		33 (37.9)	14 (31.8)	
	Agree	19 (28.8)	20 (30.8)		25 (28.7)	14 (31.8)	
	Strongly agree	8 (12.1)	11 (16.9)		8 (9.2)	11 (25)	

(n: frequency; %: percentage). The statistically significant *p* values are shown in bold (*p < 0.05)

When evaluated by gender, significant differences were found in perceptions of the sufficiency of preclinical training on tooth preparation procedures (*p* = 0.024) and impression procedures for fixed prostheses (*p* = 0.008). Male students reported higher self-perceived knowledge regarding intraoral procedures, accurate impression procedures, proper occlusion, and esthetics in removable partial denture fabrication than female students (*p* < 0.001) (Table 2).

**Fig. 1:**
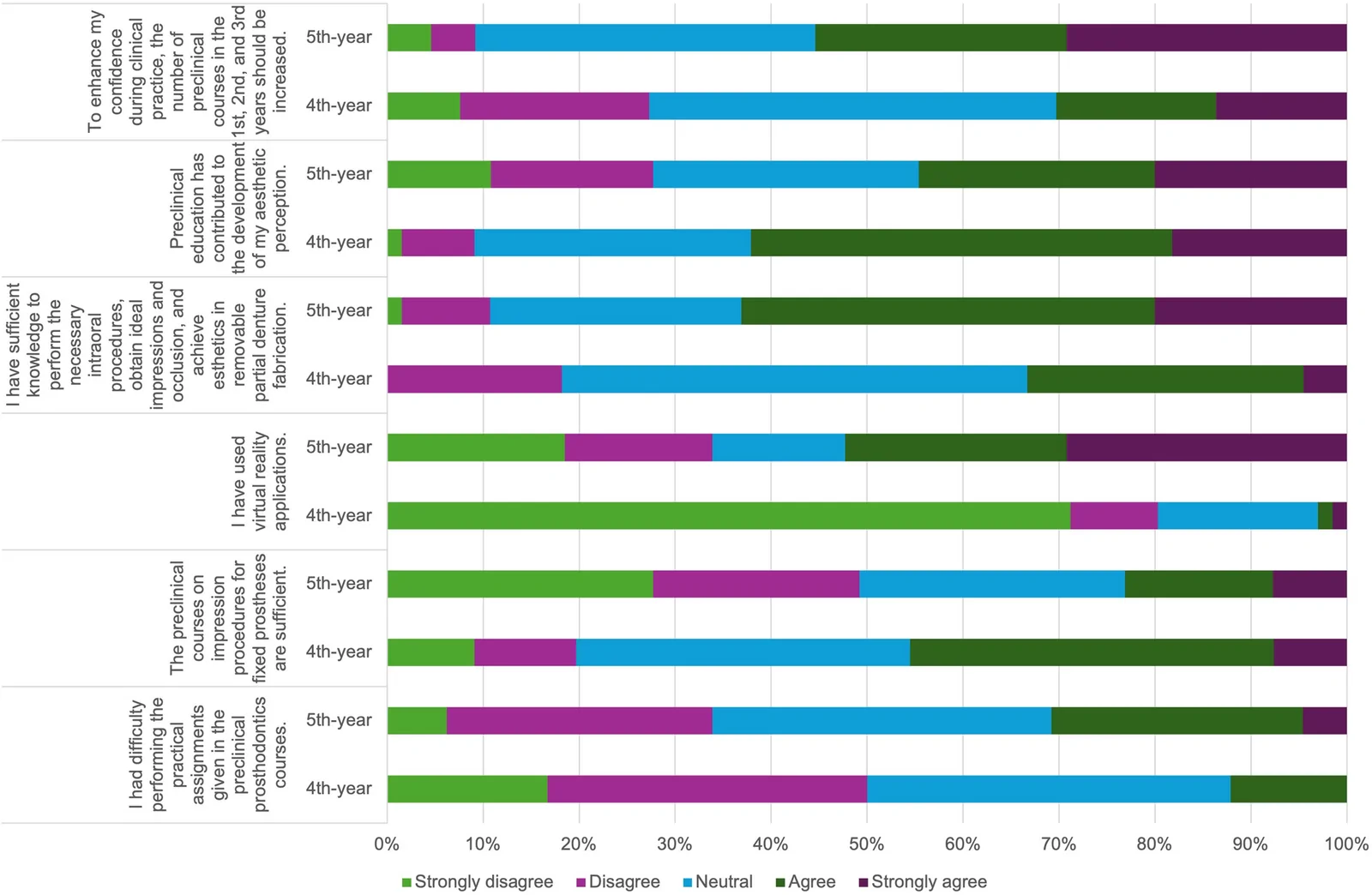
Distribution of Likert-scale responses for selected questionnaire items according to academic year. Percentage distribution of responses among 4th- and 5th-year students for selected questionnaire items related to prosthodontic education, curriculum adequacy, and clinical confidence

**Fig. 2:**
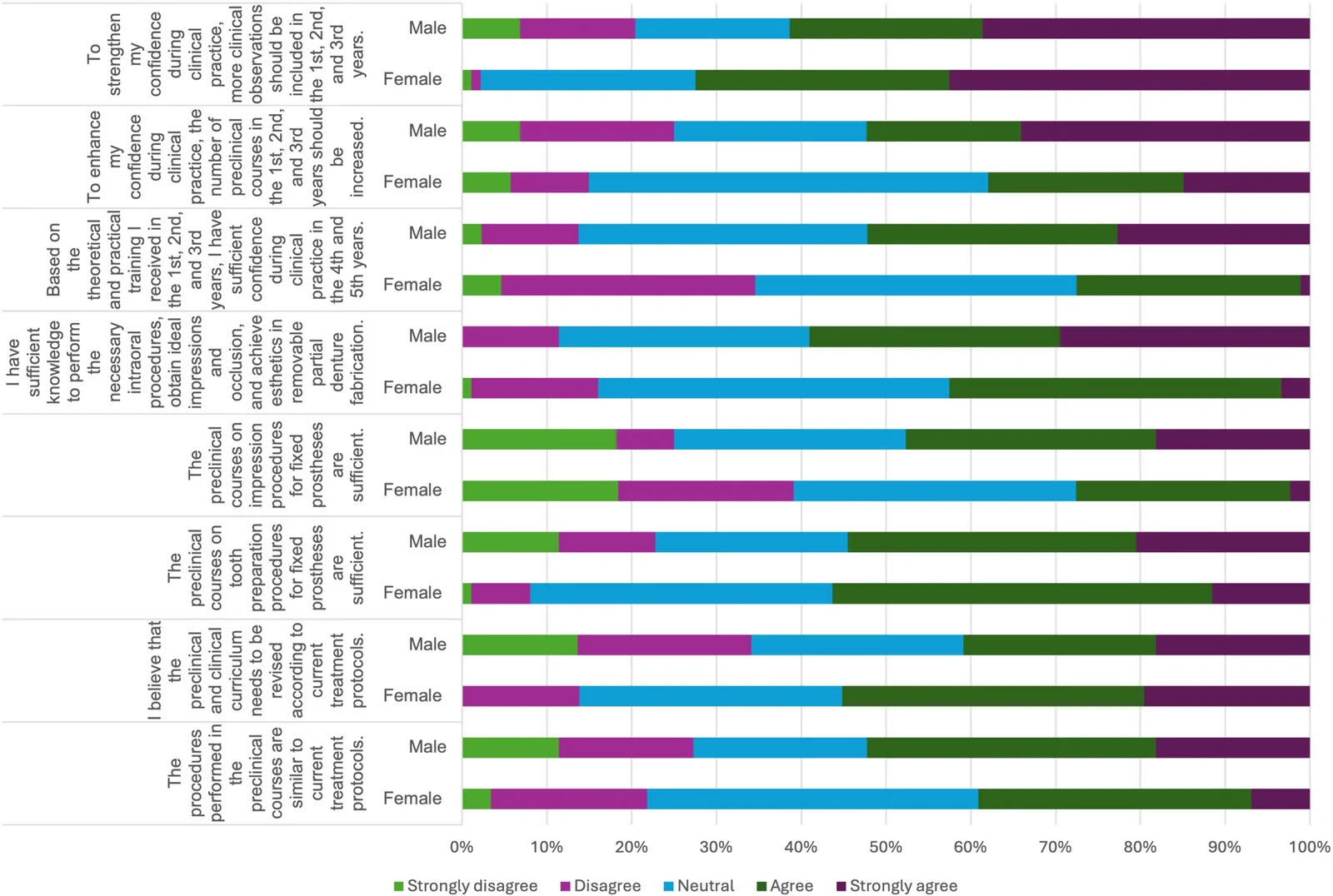
Distribution of Likert-scale responses according to gender. Percentage distribution of responses among female and male students for selected questionnaire items related to prosthodontic education, curriculum adequacy, and clinical confidence

In the questions related to clinical confidence and learning needs, statistically significant differences were found according to both academic year and gender, as shown in Table 3. When evaluated by academic year, 5th-year students more frequently reported the need to increase preclinical courses in the 1st, 2nd, and 3rd years to enhance confidence during clinical practice (*p* = 0.017). When evaluated by gender, male students reported higher self-confidence during clinical practice based on the theoretical and practical training received in the 1st, 2nd, and 3rd years (*p* < 0.001). Significant gender differences were also observed regarding the perceived need to increase preclinical courses (*p* = 0.019) and include more clinical observations in the early years of dental education (*p* = 0.012) (Table 3). The distributions of statistically significant responses according to academic year and gender are presented in Figs. 1 and 2, respectively.

**Table 3:** Comparison of students’ responses to the questionnaire items related to clinical confidence and learning needs according to academic year and gender

Questions	Responses	4th-year	5th-year	*p*	Female	Male	*p*
		*n* (%)	*n* (%)		*n* (%)	*n* (%)	
Based on the theoretical and practical training I received in the 1st, 2nd, and 3rd years, I have sufficient confidence during clinical practice in the 4th and 5th years.	Strongly disagree	2 (3)	3 (4.6)	0.285	4 (4.6)	1 (2.3)	< 0.001*
	Disagree	16 (24.2)	15 (23.1)		26 (29.9)	5 (11.4)	
	Neutral	29 (43.9)	19 (29.2)		33 (37.9)	15 (34.1)	
	Agree	16 (24.2)	20 (30.8)		23 (26.4)	13 (29.5)	
	Strongly agree	3 (4.5)	8 (12.3)		1 (1.1)	10 (22.7)	
To improve my confidence during clinical practice, more patient and treatment videos should be included in the theoretical courses of the 1st, 2nd, and 3rd years.	Strongly disagree	1 (1.5)	1 (1.5)	0.058	0 (0)	2 (4.5)	0.247
	Disagree	6 (9.1)	0 (0)		3 (3.4)	3 (6.8)	
	Neutral	18 (27.3)	14 (21.5)		20 (23)	12 (27.3)	
	Agree	16 (24.2)	24 (36.9)		29 (33.3)	11 (25)	
	Strongly agree	25 (37.9)	26 (40)		35 (40.2)	16 (36.4)	
To enhance my confidence during clinical practice, the number of preclinical courses in the 1st, 2nd, and 3rd years should be increased.	Strongly disagree	5 (7.6)	3 (4.6)	0.017*	5 (5.7)	3 (6.8)	0.019*
	Disagree	13 (19.7)	3 (4.6)		8 (9.2)	8 (18.2)	
	Neutral	28 (42.4)	23 (35.4)		41 (47.1)	10 (22.7)	
	Agree	11 (16.7)	17 (26.2)		20 (23)	8 (18.2)	
	Strongly agree	9 (13.6)	19 (29.2)		13 (14.9)	15 (34.1)	
To strengthen my confidence during clinical practice, more clinical observations should be included in the 1st, 2nd, and 3rd years.	Strongly disagree	0 (0)	4 (6.2)	0.062	1 (1.1)	3 (6.8)	0.012*
	Disagree	4 (6.1)	3 (4.6)		1 (1.1)	6 (13.6)	
	Neutral	20 (30.3)	10 (15.4)		22 (25.3)	8 (18.2)	
	Agree	19 (28.8)	17 (26.2)		26 (29.9)	10 (22.7)	
	Strongly agree	23 (34.8)	31 (47.7)		37 (42.5)	17 (38.6)	
Non-invasive procedures such as anamnesis, intraoral, and radiographic examinations should be practiced in the 1st, 2nd, and 3rd years to improve clinical confidence.	Strongly disagree	1 (1.5)	2 (3.1)	0.764	2 (2.3)	1 (2.3)	0.592
	Disagree	2 (3)	3 (4.6)		2 (2.3)	3 (6.8)	
	Neutral	18 (27.3)	12 (18.5)		18 (20.7)	12 (27.3)	
	Agree	12 (18.2)	12 (18.5)		17 (19.5)	7 (15.9)	
	Strongly agree	33 (50)	36 (55.4)		48 (55.2)	21 (47.7)	

(n: frequency; %: percentage). The statistically significant *p* values are shown in bold (*p < 0.05)

**Fig. 3:**
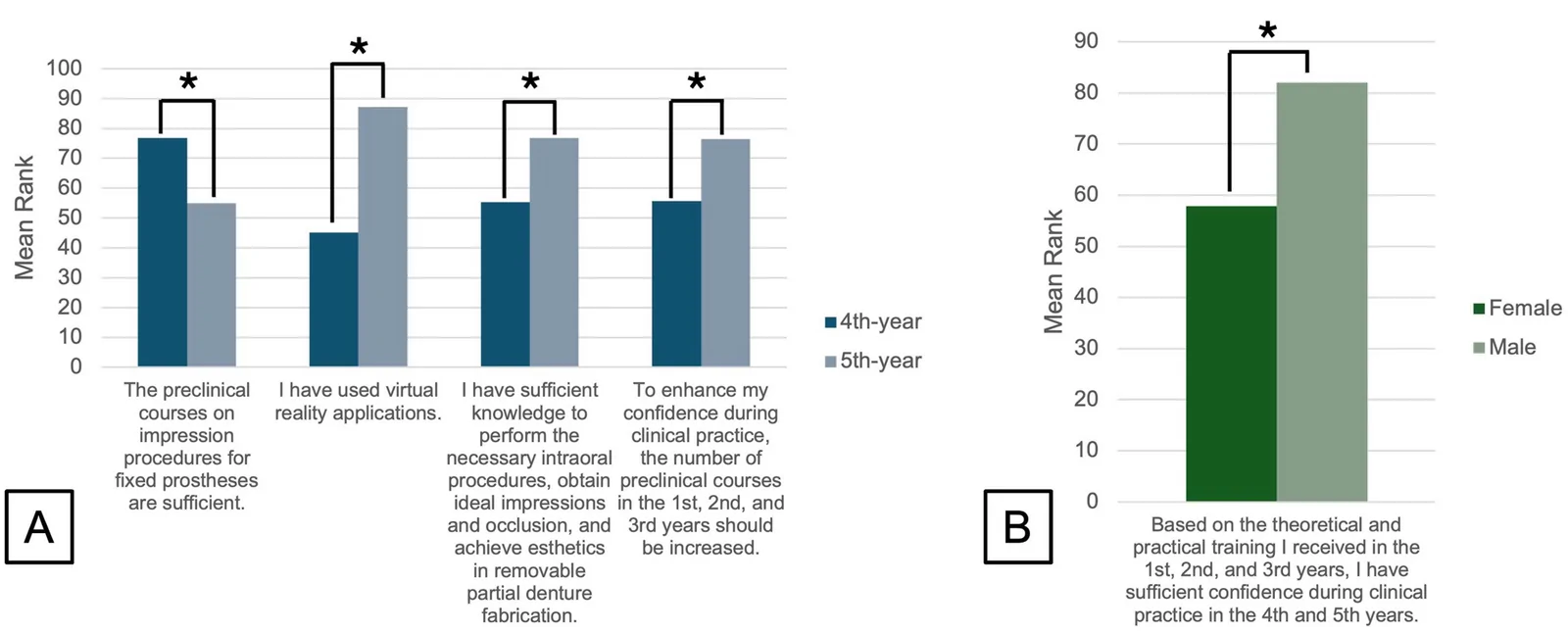
Significant differences in questionnaire responses according to academic year and gender. Mean rank values obtained from Mann–Whitney U analyses with Bonferroni correction are shown. **A** significant differences according to academic year. **B** significant difference according to gender. * *p* < 0.05 indicates statistical significance

To further account for multiple comparisons and the ordinal structure of the questionnaire responses, Mann–Whitney U analyses with Bonferroni correction were additionally performed. These analyses showed that, when evaluated by academic year, 5th-year students reported significantly lower perceived sufficiency of preclinical training on impression procedures for fixed prostheses compared with 4th-year students (*p* = 0.017). Fifth-year students reported significantly greater experience with virtual reality applications (*p* < 0.001), higher self-perceived knowledge regarding intraoral procedures, ideal impression procedures, occlusion, and esthetics in removable partial denture fabrication (*p* = 0.015), and a greater need for increasing preclinical courses in the 1st, 2nd, and 3rd years to enhance confidence during clinical practice (*p* = 0.028). When evaluated by gender, male students reported significantly higher clinical confidence based on the theoretical and practical training received in the 1st, 2nd, and 3rd years compared with female students (*p* = 0.008) (Table 4; Fig. 3).

**Table 4:** Mann–Whitney U test results with Bonferroni-adjusted p values for Likert-scale questionnaire items according to academic year and gender

Questions		4th-year MR	5th-year MR	*p*	Female MR	Male MR	*p*
1	I had difficulty performing the practical assignments given in the preclinical prosthodontics courses.	57.34	74.79	0.143	66.99	64.05	1.000
2	The procedures performed in the preclinical courses are similar to current treatment protocols.	71.20	60.72	1.000	64.03	69.90	1.000
3	The procedures performed in the clinical practice are similar to current treatment protocols.	70.97	60.95	1.000	63.32	71.30	1.000
4	I believe that the preclinical and clinical curriculum needs to be revised according to current treatment protocols.	60.64	71.45	1.000	70.67	56.76	0.975
5	I believe that the current clinical training is sufficient for professional practice after graduation.	68.35	63.62	0.504	62.60	72.72	1.000
6	The fixed prosthodontic procedures performed in preclinical courses are similar to those performed in clinical practice.	67.36	64.62	1.000	62.30	73.32	1.000
7	The preclinical courses on tooth preparation procedures for fixed prostheses are sufficient.	71.40	60.52	1.000	66.76	64.50	1.000
8	The preclinical courses on impression procedures for fixed prostheses are sufficient.	76.83	55.01	0.017*	60.75	76.39	0.522
9	The clinical procedures for fixed prosthodontic treatments are similar to current treatment protocols.	71.05	60.87	1.000	62.33	73.26	1.000
10	I have received adequate theoretical training on digital impression and workflow	66.20	65.80	1.000	62.94	72.05	1.000
11	I have had experience with digital impression procedures during clinical practice.	67.35	64.63	1.000	61.22	75.44	0.638
12	I have used virtual reality applications.	45.16	87.16	< 0.001*	64.74	68.49	1.000
13	I have found the use of virtual reality applications beneficial for chairside procedures.	65.29	66.72	1.000	68.26	61.52	1.000
14	I have sufficient knowledge about shade selection in clinical practice.	64.49	67.53	1.000	65.45	67.08	1.000
15	Preclinical education has contributed to the development of my aesthetic perception.	72.24	59.66	1.000	66.44	65.14	1.000
16	The removable partial denture procedures performed in preclinical courses are similar to those performed in clinical practice.	65.58	66.42	1.000	60.08	77.70	0.234
17	I have sufficient knowledge to plan appropriate treatments and fabricate removable partial dentures for partially edentulous patients.	58.83	73.28	0.550	62.10	73.70	1.000
18	I have sufficient knowledge to perform the necessary intraoral procedures, obtain ideal impressions and occlusion, and achieve esthetics in removable partial denture fabrication.	55.34	76.82	0.015*	60.00	77.86	0.175
19	The complete denture procedures performed in preclinical courses are similar to those performed in clinical practice.	63.92	68.11	1.000	60.21	77.45	0.255
20	Based on the theoretical and practical training I received in the 1st, 2nd, and 3rd years, I have sufficient confidence during clinical practice in the 4th and 5th years.	62.34	69.72	1.000	57.91	82.00	0.008*
21	To improve my confidence during clinical practice, more patient and treatment videos should be included in the theoretical courses of the 1st, 2nd, and 3rd years.	62.02	70.04	1.000	68.63	60.80	1.000
22	To enhance my confidence during clinical practice, the number of preclinical courses in the 1st, 2nd, and 3rd years should be increased.	55.74	76.42	0.028*	63.47	71.00	1.000
23	To strengthen my confidence during clinical practice, more clinical observations should be included in the 1st, 2nd, and 3rd years.	62.06	70.00	1.000	69.05	59.98	1.000
24	Non-invasive procedures such as anamnesis, intraoral, and radiographic examinations should be practiced in the 1st, 2nd, and 3rd years to improve clinical confidence.	64.30	67.72	1.000	68.43	61.19	1.000

Bonferroni-adjusted p values were calculated separately for academic year and gender comparisons. Sta The statistically significant *p* values are shown in bold (*p < 0.05)

*MR *mean rank

Effect size analysis showed that the statistically significant associations identified using Chi-square and Fisher’s exact tests generally had small-to-moderate effect sizes, with Cramer’s V values ranging from 0.275 to 0.333. For the additional Mann–Whitney U analyses, effect sizes ranged from small to large among the statistically significant findings. The largest effect size was observed for the difference between academic years in the use of virtual reality applications (*r* = 0.584), while the remaining significant Mann–Whitney U findings showed small-to-moderate effect sizes (*r* = 0.284–0.313).

When examining the association between the statement “I believe that the preclinical and clinical curriculum needs to be revised according to current treatment protocols” and the statements “The procedures performed in the preclinical courses are similar to current treatment protocols” and “The procedures performed in the clinical practice are similar to current treatment protocols,” a statistically significant relationship was observed (*p* < 0.001) (Table 5).

**Table 5:** Association between the perceived need for curriculum revision and students’ perceptions of the similarity of preclinical and clinical procedures to current treatment protocols

		I believe that the preclinical and clinical curriculum needs to be revised according to current treatment protocols.					
	Responses	Strongly disagree	Disagree	Neutral	Agree	Strongly agree	
		*n*(%)	*n*(%)	*n*(%)	*n*(%)	*n*(%)	*p*
The procedures performed in the preclinical courses are similar to current treatment protocols.	Strongly disagree	0(0)	0(0)	0(0)	4(9.8)	4(16)	< 0.001*
	Disagree	0(0)	0(0)	8(21.1)	8(19.5)	7(28)	
	Neutral	0(0)a	5(23.8)	14(36.8)	14(34.1)	10(40)	
	Agree	2(33.3)	13(61.9)	14(36.8)	11(26.8)	3(12)	
	Strongly agree	4(66.7)	3(14.3)	2(5.3)	4(9.8)	1(4)	
The procedures performed in the clinical practice are similar to current treatment protocols.	Strongly disagree	0(0)	0(0)	0(0)a	3(7,3)	2(8)	< 0.001*
	Disagree	0(0)	1(4.8)	5(13.2)	6(14.6)	3(12)	
	Neutral	0(0)	2(9.5)	17(44.7)	15(36.6)	11(44)	
	Agree	1(16.7)	13(61.9)	11(28.9)	14(34.1)	6(24)	
	Strongly agree	5(83.3)	5(23.8)	5(13.2)	3(7.3)	3(12)	

(n: frequency; %: percentage). The statistically significant p values are shown in bold (*p < 0.05)

When examining the association between the statement “I believe that the current clinical training is sufficient for professional practice after graduation” and the statements “The procedures performed in the preclinical courses are similar to current treatment protocols” and “The procedures performed in the clinical practice are similar to current treatment protocols,” a statistically significant relationship was observed, as shown in Table 6. Students who perceived preclinical and clinical practices as similar to current treatment protocols reported higher self-perceived clinical competence after graduation, whereas those who perceived these practices as less aligned with current treatment protocols reported lower levels of perceived competence (*p* < 0.001) (Table 6).

**Table 6:** Association between perceived sufficiency of current clinical training for professional practice after graduation and students’ perceptions of the similarity of preclinical and clinical procedures to current treatment protocols

		I believe that the current clinical training is sufficient for professional practice after graduation.					
		Strongly disagree	Disagree	Neutral	Agree	Strongly agree	
		*n*(%)	*n*(%)	*n*(%)	*n*(%)	*n*(%)	*p*
The procedures performed in the preclinical courses are similar to current treatment protocols.	Strongly disagree	4(33.3)	4(11.4)	0(0)b	0(0)	0(0)	< 0.001*
	Disagree	2(16.7)	10(28.6)	9(18,4)	1(4.2)	1(9.1)	
	Neutral	4(33.3)	14(40)	21(42.9)	3(12.5)	1(9.1)	
	Agree	2(16.7)	6(17.1)	16(32.7)	14(58.3)	5(45.5)	
	Strongly agree	0(0)	1(2.9)	3(6.1)	6(25)	4(36.4)	
The procedures performed in the clinical practice are similar to current treatment protocols.	Strongly disagree	2(16.7)	2(5.7)	1(2)	0(0)	0(0)	< 0.001*
	Disagree	2(16.7)	7(20)	4(8.2)	0(0)	2(18.2)	
	Neutral	5(41.7)	13(37.1)	23(46.9)	3(12.5)	1(9.1)	
	Agree	3(25)	11(31.4)	16(32.7)	12(50)	3(27.3)	
	Strongly agree	0(0)	2(5.7)	5(10.2)	9(37.5)	5(45.5)	

(n: frequency; %: percentage). The statistically significant p values are shown in bold (*p < 0.05)

When examining the association between the statement “To enhance my confidence during clinical practice, the number of preclinical courses in the 1st, 2nd, and 3rd years should be increased” and the statements “The preclinical courses on tooth preparation procedures for fixed prostheses are sufficient” and “The preclinical courses on impression procedures for fixed prostheses are sufficient,” a statistically significant relationship was observed, as shown in Table 7. Students who evaluated preclinical training as sufficient reported a lower perceived need for increasing the number of preclinical courses, whereas those who perceived preclinical training as insufficient reported a greater need for more preclinical practice in the early years to support their clinical confidence (*p* < 0.001) (Table 7).

**Table 7:** Association between the perceived need to increase preclinical courses to enhance clinical confidence and students’ perceptions of the sufficiency of preclinical tooth preparation and impression training for fixed prostheses

		To enhance my confidence during clinical practice, the number of preclinical courses in the 1st, 2nd, and 3rd years should be increased.					
		Strongly disagree	Disagree	Neutral	Agree	Strongly agree	
		*n*(%)	*n*(%)	*n*(%)	*n*(%)	*n*(%)	*p*
The preclinical courses on tooth preparation procedures for fixed prostheses are sufficient.	Strongly disagree	1(12.5)	1(6.3)	0(0)	0(0)	4(14.3)	< 0.001*
	Disagree	0(0)	2(12.5)	5(9.8)	1(3.6)	3(10.7)	
	Neutral	1(12.5)	1(6.3)	25(49)	9(32.1)	5(17.9)	
	Agree	3(37.5)	7(43.8)	18(35.3)	17(60.7)	9(32.1)	
	Strongly agree	3(37.5)	5(31.3)	3(5.9)	1(3.6)	7(25)	
The preclinical courses on impression procedures for fixed prostheses are sufficient.	Strongly disagree	2(25)	4(25)	6(11.8)	3(10.7)	9(32.1)	< 0.001*
	Disagree	1(12.5)	0(0)	7(13.7)	6(21.4)	7(25)	
	Neutral	3(37.5)	3(18.8)	26(51)	7(25)	2(7.1)	
	Agree	2(25)	8(50)	10(19.6)	11(39.3)	4(14.3)	
	Strongly agree	0(0)	1(6.3)	2(3.9)	1(3.6)	6(21.4)	

(n: frequency; %: percentage). The statistically significant p values are shown in bold (*p < 0.05)

## Discussion

As in all disciplines of health sciences, the needs and methods in dentistry have changed considerably over time with advancing technology. Diagnostic methods, treatment approaches, and clinical technologies are continuously evolving compared to previous decades, and education continues to develop through the integration of contemporary technologies into fundamental training [12]. Therefore, it is essential that dental education curricula be updated and aligned with changing needs and technological progress [13]. The main objective of dental education is to support students in becoming clinically prepared and confident, with the ability to apply theoretical knowledge to practical clinical situations [11].

In the present study, significant differences were observed in students’ perceptions of their educational experiences based on both academic year and gender. When evaluated by academic year, 4th-year students perceived the contribution of preclinical education more positively, whereas 5th-year students showed a more critical perspective regarding its role in supporting self-perceived clinical competence. This finding may be attributed to the fact that 5th-year students, through their clinical internship, may evaluate preclinical education from a more clinically oriented perspective. Sampaio and Fernandez (2020) reported that as the academic level of dental students increases, their confidence in performing prosthodontic procedures improves, whereas their confidence in preclinical tasks that are rarely performed in the clinical environment decreases [7]. This finding is consistent with the results of the present study, suggesting that although 5th-year students may report greater confidence with increased clinical exposure, they also tend to adopt a more critical perspective toward preclinical training.

In addition, the higher rate of agreement among 5th-year students with the need to increase preclinical courses suggests that their clinical experience may have made them more aware of the role of preclinical education in supporting self-perceived clinical confidence and competence. Therefore, greater clinical exposure may be associated with higher self-reported confidence as well as increased awareness and critical evaluation of educational content. These findings are also consistent with those reported by Puryer et al. [6], who conducted a study among 3rd- to 5th-year dental students at the University of Bristol. Their results indicated that students’ confidence in prosthodontic procedures increased with academic progression and that the frequency of clinical practice played a key role in developing clinical confidence [6]. Similarly, in the present study, students with greater clinical experience reported higher confidence; however, their awareness of the adequacy of preclinical training and the need for curriculum improvement also appeared to increase in parallel.

According to gender, the findings revealed some differences in students’ perceptions of their educational experience. Male students perceived both preclinical and clinical practices as being more similar to current treatment protocols and generally reported higher self-perceived confidence in clinical settings. However, this finding should be interpreted cautiously, as self-confidence does not necessarily reflect objectively measured clinical ability. Therefore, the observed gender difference should be considered as a difference in perceived confidence rather than actual clinical competence. These findings should not be interpreted as inherent gender-based differences in clinical ability. Rather, they may reflect differences in self-assessment tendencies, prior learning experiences, expectations from the curriculum, or sociocultural factors influencing confidence reporting. Female students, on the other hand, agreed at a higher rate than male students with the statements indicating that the curriculum should be revised in line with current requirements, that the content of preclinical training does not fully meet clinical demands, and that more clinical observation should be included in the early years of education. These findings may suggest that, in the present sample, female students reported a more critical perspective on the scope and practical integration of the educational program and on the need to support self-perceived clinical competence. Bains et al. reported that students emphasized the need for curriculum revision according to current clinical requirements and expressed a demand for earlier clinical exposure [9]. This interpretation is further supported by Shigli et al., who reported that both students and prosthodontic faculty emphasized the need for early clinical exposure to facilitate students’ understanding of preclinical prosthodontic topics [3]. Taken together, these findings suggest that the transition from preclinical to clinical education is not limited to technical skill development but also involves professional awareness, self-evaluation, and perceived preparedness for clinical practice [6, 14]. The fundamental knowledge and manual skills acquired during the preclinical phase may support confidence in the clinical stage, while increasing clinical experience may be associated with students’ critical thinking, self-assessment, and patient-centered approach [5, 6]. This process represents not only a period of technical skill development, but also a stage of professional awareness in which students begin to critically evaluate their clinical confidence and self-perceived competence [2, 6, 15].

The findings of the present study should also be considered in relation to possible institutional, educational, and cultural factors. Differences in students’ perceptions may be influenced by the structure of the local prosthodontic curriculum, the timing and extent of clinical exposure, the availability of clinical cases, and access to digital technologies or simulation-based learning opportunities. In addition, institutional teaching methods, assessment strategies, and feedback mechanisms may affect how students evaluate the adequacy of their preclinical and clinical training. Cultural and social factors may also influence how students express self-confidence and evaluate their own preparedness for clinical practice. Therefore, the present findings should be interpreted within the specific educational context of the institution and may not be directly generalizable to dental schools with different curricula or training environments.

The increasing integration of digital technologies into contemporary dental practice highlights the need to include digital dentistry-related content in prosthodontic education [16]. In the present study, students reported limited clinical experience with digital impression procedures despite receiving theoretical instruction on digital impression and workflow. This finding may indicate a gap between theoretical exposure and practical application, consistent with previous literature suggesting that students may benefit from applying digital workflows in clinical or simulation-based settings [16, 17]. However, because the questionnaire included only a limited number of items directly related to digital workflows, these findings should be interpreted cautiously. Future studies specifically focusing on digital dentistry education are needed to better evaluate students’ practical experience, self-reported confidence, and learning needs in this area.

Preclinical education may support students’ self-perceived clinical competence, while early practical experiences may help strengthen self-reported confidence [6]. The literature also emphasizes that preclinical education is a fundamental stage for developing the manual skills required for performing clinical procedures [2]. However, factors such as the duration of preclinical education and the frequency of practice sessions have been reported to be associated with students’ confidence levels and stress. Therefore, the scope and structure of preclinical education may be related not only to students’ self-reported clinical confidence but also to their self-assessment skills and professional awareness [6, 14]. In this context, students’ confidence should be interpreted as part of a broader learning process in which they gradually evaluate their own preparedness, recognize their limitations, and adjust their expectations according to clinical experience. Greater exposure to real or simulated clinical situations may help students compare their perceived abilities with the practical demands of clinical practice, thereby supporting more realistic self-evaluation [4]. Accordingly, structuring preclinical and clinical training as complementary components and strengthening practice-based learning strategies may support students’ perceived preparedness, professional confidence, and clinical perspective [2].

Although several statistically significant differences were observed, these findings should be interpreted cautiously, as statistical significance does not necessarily indicate a meaningful educational impact. Therefore, the practical relevance of the findings should be considered together with effect size values and the broader educational context.

The limitations of this study should be considered when interpreting the findings. As the study was conducted at a single dental faculty with 131 participants, the results are context-dependent and may have limited generalizability to institutions with different curricula or clinical training models. The cross-sectional design and absence of longitudinal follow-up restrict causal interpretation and the evaluation of changes in students’ perceptions over time. In addition, the use of a self-reported questionnaire may introduce response, recall, and social desirability biases, and the study assessed perceived rather than objectively measured clinical competence. The content validity finding should also be interpreted cautiously because the Lawshe assessment was based on a five-member expert panel. Future multicenter and longitudinal studies with larger samples, objective competency assessments, educational intervention designs, digital dentistry competency measurements, and analyses comparing perceived confidence with actual clinical performance are needed.

## Conclusion

Overall, the findings of this study suggest that dental students’ perceptions of prosthodontic education differ according to academic year and gender. Fifth-year students tended to evaluate certain aspects of preclinical training more critically, particularly impression procedures for fixed prostheses, while also reporting greater experience with virtual reality applications, higher self-perceived knowledge in selected removable partial denture procedures, and a greater perceived need for increased preclinical training. Male students reported higher self-confidence during clinical practice based on their earlier theoretical and practical training; however, this finding should be interpreted as self-reported confidence rather than objective clinical competence.

These findings suggest that closer alignment between preclinical and clinical prosthodontic education, integration of updated clinical procedures, and expanded opportunities for early clinical exposure may help support students’ perceived preparedness, clinical confidence, and self-evaluation. However, because this was a single-center, cross-sectional, and perception-based study, curriculum-related implications should be interpreted cautiously and should not be considered definitive recommendations for curriculum restructuring. Future multicenter and longitudinal studies incorporating objective competency assessments, educational intervention designs, digital dentistry competency measurements, and comparisons between perceived confidence and actual clinical performance are needed.

## Supplementary Information

Supplementary Material 1.

## Data Availability

The datasets used and/or analysed during the current study are available from the corresponding author on reasonable request.
