# Supplementary material for: Dental students’ perceptions of preclinical and clinical prosthodontic education: a cross-sectional survey
Source: BMC Med Educ. 2026 Jul 8;26:1416. doi: 10.1186/s12909-026-09751-1 (PMC13540830; doi:10.1186/s12909-026-09751-1)
Supplement: Supplementary file 1 [file 12909_2026_9751_MOESM1_ESM.docx]

| Questions  Supplementary Material 1. Questionnaire Used in the Study | | Responses |
| --- | --- | --- |
| 1. | I had difficulty performing the practical assignments given in the preclinical prosthodontics courses. | Strongly disagree |
|  |  | Disagree |
|  |  | Neutral |
|  |  | Agree |
|  |  | Strongly agree |
| 2. | The procedures performed in the preclinical courses are similar to current treatment protocols. | Strongly disagree |
|  |  | Disagree |
|  |  | Neutral |
|  |  | Agree |
|  |  | Strongly agree |
| 3. | The procedures performed in the clinical practice are similar to current treatment protocols. | Strongly disagree |
|  |  | Disagree |
|  |  | Neutral |
|  |  | Agree |
|  |  | Strongly agree |
| 4. | I believe that the preclinical and clinical curriculum needs to be revised according to current treatment protocols. | Strongly disagree |
|  |  | Disagree |
|  |  | Neutral |
|  |  | Agree |
|  |  | Strongly agree |
| 5. | I believe that the current clinical training is sufficient for professional practice after graduation. | Strongly disagree |
|  |  | Disagree |
|  |  | Neutral |
|  |  | Agree |
|  |  | Strongly agree |
| 6. | The fixed prosthodontic procedures performed in preclinical courses are similar to those performed in clinical practice. | Strongly disagree |
|  |  | Disagree |
|  |  | Neutral |
|  |  | Agree |
|  |  | Strongly agree |
| 7. | The preclinical courses on tooth preparation procedures for fixed prostheses are sufficient. | Strongly disagree |
|  |  | Disagree |
|  |  | Neutral |
|  |  | Agree |
|  |  | Strongly agree |
| 8. | The preclinical courses on impression procedures for fixed prostheses are sufficient. | Strongly disagree |
|  |  | Disagree |
|  |  | Neutral |
|  |  | Agree |
|  |  | Strongly agree |
| 9. | The clinical procedures for fixed prosthodontic treatments are similar to current treatment protocols. | Strongly disagree |
|  |  | Disagree |
|  |  | Neutral |
|  |  | Agree |
|  |  | Strongly agree |
| 10. | I have received adequate theoretical training on digital impression and workflow. | Strongly disagree |
|  |  | Disagree |
|  |  | Neutral |
|  |  | Agree |
|  |  | Strongly agree |
| 11. | I have had experience with digital impression procedures during clinical practice. | Strongly disagree |
|  |  | Disagree |
|  |  | Neutral |
|  |  | Agree |
|  |  | Strongly agree |
| 12. | I have used virtual reality applications. | Strongly disagree |
|  |  | Disagree |
|  |  | Neutral |
|  |  | Agree |
|  |  | Strongly agree |
| 13. | I have found the use of virtual reality applications beneficial for chairside procedures. | Strongly disagree |
|  |  | Disagree |
|  |  | Neutral |
|  |  | Agree |
|  |  | Strongly agree |
| 14. | I have sufficient knowledge about shade selection in clinical practice. | Strongly disagree |
|  |  | Disagree |
|  |  | Neutral |
|  |  | Agree |
|  |  | Strongly agree |
| 15. | Preclinical education has contributed to the development of my aesthetic perception. | Strongly disagree |
|  |  | Disagree |
|  |  | Neutral |
|  |  | Agree |
|  |  | Strongly agree |
| 16. | The removable partial denture procedures performed in preclinical courses are similar to those performed in clinical practice. | Strongly disagree |
|  |  | Disagree |
|  |  | Neutral |
|  |  | Agree |
|  |  | Strongly agree |
| 17. | I have sufficient knowledge to plan appropriate treatments and fabricate removable partial dentures for partially edentulous patients. | Strongly disagree |
|  |  | Disagree |
|  |  | Neutral |
|  |  | Agree |
|  |  | Strongly agree |
| 18. | I have sufficient knowledge to perform the necessary intraoral procedures, obtain ideal impressions and occlusion, and achieve esthetics in removable partial denture fabrication. | Strongly disagree |
|  |  | Disagree |
|  |  | Neutral |
|  |  | Agree |
|  |  | Strongly agree |
| 19. | The complete denture procedures performed in preclinical courses are similar to those performed in clinical practice. | Strongly disagree |
|  |  | Disagree |
|  |  | Neutral |
|  |  | Agree |
|  |  | Strongly agree |
| 20. | Based on the theoretical and practical training I received in the 1st, 2nd, and 3rd years, I have sufficient confidence during clinical practice in the 4th and 5th years. | Strongly disagree |
|  |  | Disagree |
|  |  | Neutral |
|  |  | Agree |
|  |  | Strongly agree |
| 21. | To improve my confidence during clinical practice, more patient and treatment videos should be included in the theoretical courses of the 1st, 2nd, and 3rd years. | Strongly disagree |
|  |  | Disagree |
|  |  | Neutral |
|  |  | Agree |
|  |  | Strongly agree |
| 22. | To enhance my confidence during clinical practice, the number of preclinical courses in the 1st, 2nd, and 3rd years should be increased. | Strongly disagree |
|  |  | Disagree |
|  |  | Neutral |
|  |  | Agree |
|  |  | Strongly agree |
| 23. | To strengthen my confidence during clinical practice, more clinical observations should be included in the 1st, 2nd, and 3rd years. | Strongly disagree |
|  |  | Disagree |
|  |  | Neutral |
|  |  | Agree |
|  |  | Strongly agree |
| 24. | Non-invasive procedures such as anamnesis, intraoral, and radiographic examinations should be practiced in the 1st, 2nd, and 3rd years to improve clinical confidence. | Strongly disagree |
|  |  | Disagree |
|  |  | Neutral |
|  |  | Agree |
|  |  | Strongly agree |
